# Mechanisms of Change in Emulsifying Capacity Induced by Protein Denaturation and Aggregation in Quick-Frozen Pork Patties with Different Fat Levels and Freeze–Thaw Cycles

**DOI:** 10.3390/foods11010044

**Published:** 2021-12-24

**Authors:** Nan Pan, Wei Wan, Xin Du, Baohua Kong, Qian Liu, Hong Lv, Xiufang Xia, Fangfei Li

**Affiliations:** 1 College of Food Science, Northeast Agricultural University, Harbin 150030, China; pannan36@163.com (N.P.); ww2417013241@163.com (W.W.); dbnydxdx@163.com (X.D.); kongbh63@hotmail.com (B.K.); liuqian@neau.edu.cn (Q.L.); 2 Department of Food and Pharmaceuticals, Harbin Light Industry School, Harbin 150076, China; lvhongq@126.com; 3 College of Forestry, Northeast Forestry University, Harbin 150040, China

**Keywords:** emulsifying property, myofibrillar protein, protein denaturation and aggregation, quick-frozen patties, freeze–thaw cycle

## Abstract

Herein, we discuss changes in the emulsifying properties of myofibrillar protein (MP) because of protein denaturation and aggregation from quick-frozen pork patties with multiple fat levels and freeze–thaw (F–T) cycles. Protein denaturation and aggregation were confirmed by the significantly increased surface hydrophobicity, turbidity, and particle size, as well as the significantly decreased solubility and absolute zeta potential, of MPs with increases in fat levels and F–T cycles (*p* < 0.05). After multiple F–T cycles, the emulsifying activity and emulsion stability indices of all samples were significantly reduced (*p* < 0.05). The emulsion droplets of MP increased in size, and their distributions were dense and irregular. The results demonstrated that protein denaturation and aggregation due to multiple F–T cycles and fat levels changed the distribution of surface chemical groups and particle sizes of protein, thus affecting the emulsifying properties.

## 1. Introduction

Quick-frozen patties are a type of emulsion-type comminuted meat product [1]. They are usually composed of muscle proteins, fat particles, water, salt, and nonmeat ingredients combined by mincing, chopping, mixing, molding, quick freezing, and frozen storage. In the emulsified meat system, muscle proteins and fat particles are important components. For muscle proteins, myofibrillar protein (MP), which forms 55–60% of total protein, plays an important role in emulsifying properties, and more than 90% of these proteins participate in the emulsification processes [2,3]. MP acts as an emulsifier to maintain the balance of protein–fat and protein–water by adsorbing on the surface of fat globules to form the interfacial protein film [1]. Fat particles are another important ingredient for improving the quality of emulsified meat products [4]. Fat droplets are entrapped in the protein matrix and stabilized via a protein network structure [1]. The addition of fat contributes to the formation of a stable meat emulsion and the enhancement of the flavor, juiciness, and textural properties of emulsified meat products [5]. In addition to muscle proteins and fat particles, protein composition, type (such as cereal protein and whey protein), and processing conditions (such as thermal treatment [6], pH [7], mechanical stress [8], and frozen storage [9]) influence the emulsifying properties of proteins.

Quick freezing and frozen storage are the most extensively used methods for extending the shelf-life of quick-frozen patties [10]. Thawing is a common and key step before quick-frozen patties are further processed and consumed [11]. When the cold chain is unsound, temperature variation during freezing, frozen storage, thawing, and transportation can induce freeze–thaw (F–T) cycles [10,12]. During F–T cycles, the integrity of minced fat and lean meat from patties is destroyed, and some reactive oxygen species are released, which could intensify fat and protein oxidation [13]. Oxidation could induce protein conformational partial unfolding, thereby increasing the extent of exposed hydrophobic groups that were buried in the protein [14]. Furthermore, protein denaturation due to oxidative reactions and the formation of protein aggregates via hydrophobic interactions is accelerated by repeated F–T cycles [3,15].

Although the emulsification property of MP has been extensively discussed, there are few studies that have investigated the relationship among emulsifying properties, protein denaturation, and aggregation during multiple F–T cycles. Therefore, this study clarified the mechanism of decreased MP emulsification characteristics by analyzing the surface hydrophobicity and solubility and by determining the turbidity, particle size, and zeta potential of protein from patties with various fat levels.

## 2. Materials and Methods

### 2.1. Materials

Fresh pork ham and back fat were obtained from a local supermarket (Harbin, Heilongjiang, China) at 24 h postmortem. All chemicals reagents used were of analytical grade.

### 2.2. Preparation and Repeated Freeze–Thaw Treatment of Patties

Based on our previous study, various fat levels (0%, 5%, 10%, 15%, and 20%) of patties were prepared, and a procedure of F–T cycles were performed [16]. Fresh pork ham muscle was trimmed of visible fat and connective tissues using a butcher knife. Lean meat and back fat were separately cut into small chunks and ground using a meat grinder equipped with a 0.5 mm plate (Braher International, San Sebastian, Spain). There were five different formulations with combinations of lean/fat as follows: 100/0%, 95/5%, 90/10%, 85/15%, and 80/20%. During patty manufacturing, 2% NaCl and 20% ice-cold water were incorporated into the meat mixture for each fat level. Finally, patties were individually wrapped in polyethylene bags. There were 60 patties (approximately 100 g each). All patties were separated into five groups, and each of them underwent F–T cycles. Samples subjected to 1, 3, and 5 F–T cycles were used for experiments. There were three independent batches of patties, and each parameter was measured in triplicate.

### 2.3. Isolation of MP

MP extraction was performed on the patties following F–T cycles as described by Li et al. [15]. Patties were homogenized 3 times with 4 vol (*w*/*v*) cold-extracted solution (10 mM sodium phosphate, 0.1 M NaCl, 2 mM MgCl_2_, and 1 mM EGTA, pH = 7.0) for 1 min with a tissue homogenizer (Lu Shen Instrument Equipment, Shanghai, China). The mixture was centrifuged at 6500× *g* for 15 min at 4 °C. The obtained pellet was then washed three times with 4 vol of 0.1 M NaCl under the same condition as above, except that in the last wash, the myofibril suspension was filtered through four layers of cheese cloth to remove connective tissue, and its pH was adjusted to 6.0 with 0.1 M HCl prior to centrifugation. The obtained MP extractions were kept at 4 °C and used within 48 h. The biuret method was used to determine the protein concentration.

### 2.4. Determination of Protein Denaturation

#### 2.4.1. Protein Surface Hydrophobicity

Protein surface hydrophobicity was determined as per Benjakul et al. [17] using 1-anilino-8-naphthalenesulfonate (ANS) (Sigma Aldrich Chemical Co.(St. Louis, MO, USA)) as a fluorescence probe using a fluorescence spectrophotometer (F-4500, Hitachi, Tokyo, Japan). The MP solution was adjusted to 0.08, 0.16, 0.24, 0.32, and 0.4 mg/mL using 15 mM of piperazine-N,N′-bis-2-ethanesulphonic acid (PIPES) buffer (0.6 M NaCl; pH 6.25) in a total volume of 4 mL. Then, 40 μL of ANS (8 mM) was mixed with the solution under dark conditions at 25 °C for 20 min. The excitation and emission wavelengths were 370 and 470 nm, respectively, with a slit width of 5 nm. For each sample, surface hydrophobicity was determined from the initial slope of net relative fluorescence.

#### 2.4.2. Protein Solubility

Protein solubility was analyzed by the methods of Wang et al. [18]. Note that 10 mL of MP suspension (2 mg/mL) was centrifuged at 5000× *g* for 15 min at 4 °C. Protein solubility (%) is the percentage of supernatant protein concentration and protein concentration before centrifugation.

### 2.5. Determination of Protein Aggregation

#### 2.5.1. Protein Turbidity Determination

Protein turbidity was analyzed using the absorbance method according to Pan et al. [19]. The MP dispersions were dissolved in 15 mM of PIPES buffer solution (0.6 M NaCl, pH 6.25) to obtain a concentration of 1 mg/mL. The absorbance of the sample was measured at a 660 nm wavelength using a 720 G visible spectrophotometer (Shanghai Yidian Analytical Instrument Co., Ltd., Shanghai, China). Turbidity was expressed as the absorbance value.

#### 2.5.2. Particle Size Analysis

The Mastersizer 2000 (Malvern Instruments Ltd., Worcestershire, UK) was used to monitor particle size and particle size distribution by preparing MP solution. The MP dispersions were diluted to 1 mg/mL with deionized water to avoid multiple scattering. One milliliter of MP solution was added into a clear zeta cell. Size measurements were reported as the volume-weighted mean diameter (*d*_4,3_) (Equation (1)) and surface-weighted mean diameter (*d*_3,2_) (Equation (2)). The indexes of protein were analyzed using the Malvern Mastersizer software (version 5.12c, Malvern Instruments Co. Ltd., Worcestershire, UK) [15]

(1) $$d_{4,3}=\frac{\sum n_{i}d_{i}^{4}}{\sum n_{i}d_{i}^{3}}(\mu m)$$

(2) $$d_{3,2}=\frac{\sum n_{i}d_{i}^{3}}{\sum n_{i}d_{i}^{2}}(\mu m)$$

where *n_i_* is the number of droplets of diameter *d_i_*.

#### 2.5.3. Zeta Potential Analysis

Zeta potential was determined by the methods of Zhang et al. [20], with minor modifications. Protein dispersions were diluted to 1 mg/mL with deionized water to avoid the scattering effect. About 1 mL of MP solution was used for testing, and the average measurement was 6. The value was obtained by a zeta potential analyzer (Malvern Instruments Co. Ltd., Worcestershire, UK) at 25 °C and expressed in mV.

### 2.6. Emulsifying Properties

#### 2.6.1. Emulsifying Activity Index and Emulsion Stability Index 

Both emulsifying activity index (EAI) and emulsion stability index (ESI) were measured as per the method of Pearce and Kinsella [21]. Protein dispersions were diluted to 1 mg/mL with 15 mM of PIPES buffer solution (0.6 M NaCl, pH 6.25). Note that 2 mL of soy oil and 8 mL of protein solution (1 mg/mL, *w*/*v*) were homogenized at 1200 rpm for 1 min using a homogenizer blender (Model T25 D S-25, IKA, DE). Then, emulsion (50 μL) was taken at 0.5 cm from the bottom of the plastic tube and diluted with 5 mL of 0.1% sodium dodecyl sulphate (SDS) solution at 0 and 10 min after homogenization. The absorbances of different emulsions at 500 nm were measured using a UT-1800 spectrophotometer (Beijing Purkinje General Instrument Co., Ltd., Beijing, China). SDS solution (0.1%, *w*/*v*) was used as a blank. The EAI and ESI were calculated as:

(3) $$EAI(m^{2}/g)=\frac{2\times 2.303}{c\times (1-\varphi )\times 10^{4}}\times A_{0}\times dilution$$

(4) $$ESI(\%)=\frac{A_{10}}{A_{0}}\times 100$$

where c is the protein concentration before emulsification (mg/mL), φ is the oil volume fraction (*v*/*v*) of emulsion (0.25), dilution is 100, and *A*_0_ and *A*_10_ are the absorption at 0 and 10 min, respectively.

#### 2.6.2. Microstructure of Emulsion

As shown in Section 2.5.1, a small drop of emulsion was obtained and immediately observed using an optical microscope (Labomed Lx400, Labo America, Inc., Fremont, CA, USA) with objective lenses of 40×. For capturing images of the emulsion, a digital camera connected to the microscope was used. The diameter of protein emulsion droplets was analyzed using Image-pro plus (Media Cybernetics, Silver Spring, MD, USA).

### 2.7. Statistical Analysis

All experiments were performed in three replicates with triplicate measurements for each sample. All data were represented as the mean ± standard error (SE) and calculated using Statistix 8.1 software package with the General Linear Models procedure (Analytical Software, St Paul, MN, USA). Moreover, to determine significant differences among means, one-way analysis of variance (ANOVA) with Tukey’s multiple comparisons was used (*p* < 0.05). The graphs were obtained using Sigma plot 12.5. Principal component analysis (PCA) was performed among the indicators (surface hydrophobicity, protein solubility, *d*_4,3_, *d*_3,2_, zeta potential, protein turbidity, droplet diameter, EAI and ESI), as well as between the indicators and different F–T cycles, using IBM SPSS Statistics version 22.0 (Tulsa, OK, USA) to identify similarities and differences between samples.

## 3. Results and Discussion

### 3.1. Protein Denaturation

#### 3.1.1. Protein Surface Hydrophobicity

**Figure 1:**
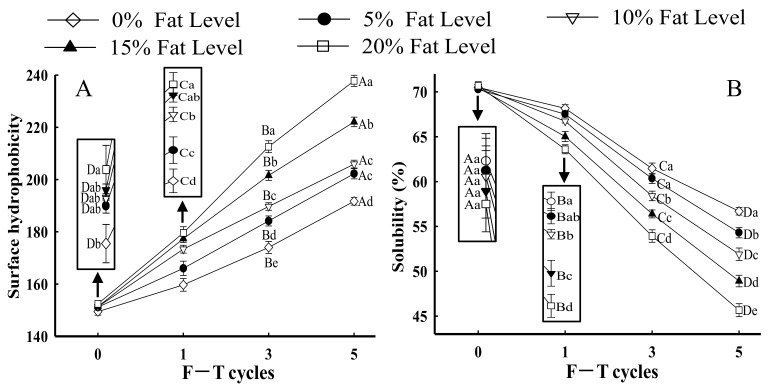
Influence of F–T cycles on surface hydrophobicity (**A**) and solubility (**B**) of myofibrillar protein from quick-frozen patties with different fat levels. Values are given as the mean ± SE. The means for the same fat levels with different uppercase letters (A to D) and the same F–T cycles with different lowercase letters (a to e) differed significantly (*p* < 0.05).

Surface hydrophobicity reflects minor changes in the chemical and physical states of proteins, which could be an important parameter for evaluating protein denaturation [22]. Moreover, surface hydrophobicity is related to the functional properties of proteins, such as solubility and emulsifying property [23]. As shown in Figure 1A, the surface hydrophobicity of MP from all samples with the same fat levels significantly increased after five F–T cycles (*p* < 0.05). The results indicated that the interaction between proteins changed from hydrophilic to hydrophobic. Generally, hydrophobic amino acids were occluded in the core of protein molecules and located in the cavities of hydrophobic binding between proteins [24,25]. With increasing F–T cycles, the structure and conformation of protein molecules were unfolded, and certain hydrophobic groups were exposed to the polar environments of the protein surface step by step, which accelerated the opening of the interior hydrophobic domain and subsequently enhanced the hydrophobicity of the protein surface [3]. 

When the F–T cycles were the same, the surface hydrophobicity of samples demonstrated a considerable increase with increased fat levels (*p* < 0.05). In particular, in samples with 20% fat at the fifth F–T cycle, surface hydrophobicity increased by 23.24% compared with samples without added fat. The results were attributed to lipid oxidation, which intensified the degree of protein oxidation. Oxidative processes could change protein conformation, thus leading to the exposure of hydrophobic groups hidden in proteins [3,16]. Similar results were obtained by Fu et al. [26], who demonstrated that there was a significant increase in the surface hydrophobicity of beef protein with increases in oxidant concentration. Furthermore, Xia et al. [27] reported that protein with a higher surface hydrophobicity could increase protein sensitivity to denaturation. The denatured MP directly affected the protein structure [28]. Riebroy et al. [29] obtained a similar result that increased surface hydrophobicity of fish protein was related to the greater exposure of hydrophobic groups, which subsequently promoted protein denaturation and subsequent aggregation in turn.

#### 3.1.2. Protein Solubility

Protein solubility, which is the most practical indicator to show protein denaturation and aggregation, refers to the ability of proteins to be dispersed in water [30,31]. As shown in Figure 1B, in samples with the same fat levels, the solubility of MP significantly decreased with increases in the number of F–T cycles (*p* < 0.05). Furthermore, in samples with 20% fat level at the fifth F–T cycle, protein solubility decreased by 34.41% compared with samples without added fat. Li et al. [32] reported that the decrease in solubility from fish protein subjected to F–T cycles might be attributed to the formation of high-molecular weight aggregates (>200 kDa). Moreover, protein solubility depends on the balance between protein–protein and protein–solvent interactions [33]. When samples were subjected to repeated freezing and thawing processes, protein conformation was partially unfolded because of hydrophobic interactions, which reduced the protein–water interaction and hence decreased protein solubility [34]. The results negatively correlated with changes in protein surface hydrophobicity (Figure 1A). Zhou and Yan [35] reported that with increase in exposure of hydrophobic groups, the solubility of proteins was reduced. Shen et al. [36] confirmed that a decline in solubility of porcine protein was accompanied by the exposure of hydrophobic groups.

With increases in fat level, the protein solubility of samples with the same F–T cycles exhibited an increasing tendency. Previously, our studies confirmed that lipid oxidation could increase the extent of protein oxidation, which led to the exposure of a large number of intra/intermolecular S–S bonds and hydrophobic groups and thus promoted protein cross-linking and the formation of insoluble aggregates [16]. Both hydrophobic regions and insoluble aggregates could result in a reduction in protein solubility. Lv et al. [37] obtained similar results in that the solubility of MP oxidized by malondialdehyde was considerably less.

### 3.2. Protein Aggregation

#### 3.2.1. Particle Size Analysis

**Figure 2:**
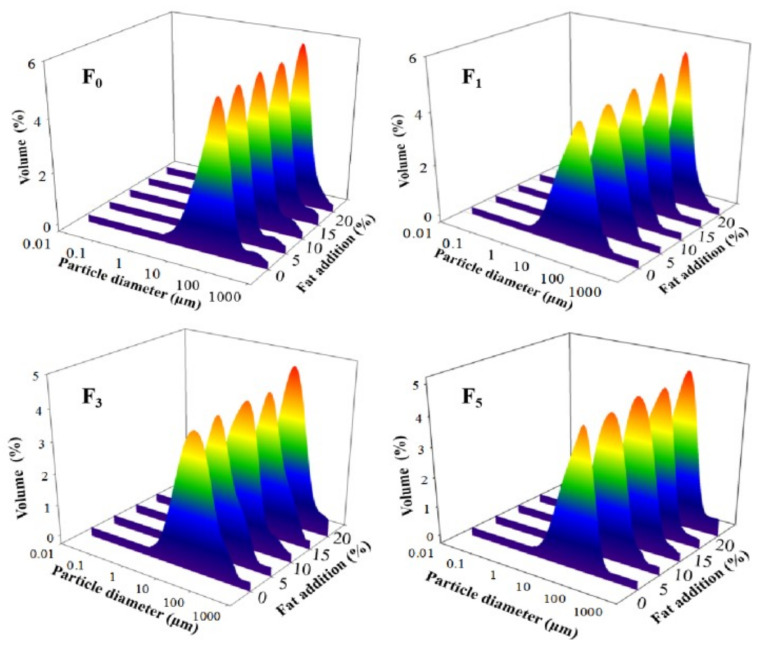
Influence of F–T cycles on volume-based particle size distribution of myofibrillar protein from quick-frozen patties with different fat levels. F_0_, F_1_, F_3_ and F_5_ means different F–T cycles.

The particle size of a protein is an important parameter for evaluating protein aggregation behavior [38]. Changes in particle size distribution and average particle size (*d*_4,3_ and *d*_3,2_) helped evaluate the particle size of proteins. As shown in Figure 2, a monomodal distribution of peaks was observed for all samples. With increases in fat levels and F–T cycles, the peak of MP samples shifted to a more extensive size range, their distribution became gradually wider, and the peak height gradually reduced. This phenomenon could be explained by the fact that fat oxidation increased the protein oxidation rate during F–T cycles, and soluble aggregates were then formed by covalent cross-linking at first. As the degree of oxidation increased, certain insoluble aggregates were formed. Thus, an extensive size range and a wide peak were identified [39]. Furthermore, with increases in the number of F–T cycles, the protein structure gradually unfolded, which subsequently participated in the processes of protein aggregation and led to a larger particle size monomodal distribution [16].

The particle sizes of *d*_4,3_ and *d*_3,2_ of MP solution significantly increased to 56.54 and 23.38 μm, respectively, as the fat level increased to 20% after five F–T cycles (*p* < 0.05) (Table 1). This indicated the occurrence of large aggregation or coalescence between proteins. The increase in particle size could be explained by the fact that protein denaturation and oxidative reaction during F–T cycles increased the generation of aggregates via intra/intermolecular disulfides and dityrosine cross-links [40,41]. Wang et al. [42] obtained similar results, showing that an increase in particle size from quick-frozen yellow croaker MP solution was attributed to the formation of protein polymers after protein denaturation and oxidation. Shen et al. [36] reported that hydrophobic interactions enhanced interactions between proteins and then produced large aggregates, which resulted in an increase in particle size.

#### 3.2.2. Zeta Potential

**Figure 3:**
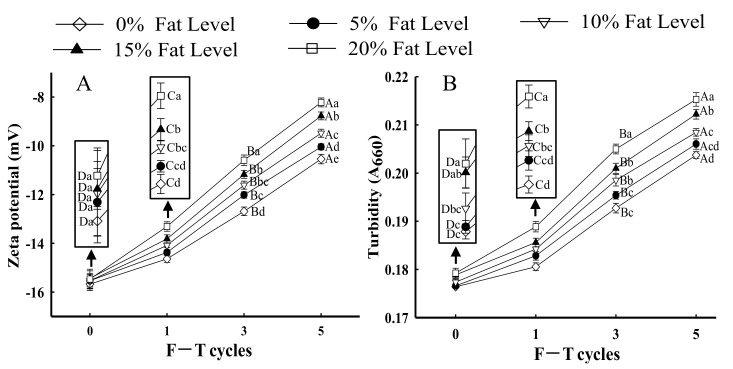
Influence of F–T cycles on zeta potential (**A**) and turbidity (**B**) of myofibrillar protein from quick-frozen patties with different fat levels. Values are given as the mean ± SE. The means for the same fat levels with different uppercase letters (A to D) and the same F–T cycles with different lowercase letters (a to e) differed significantly (*p* < 0.05).

The net surface charge of proteins can be evaluated using zeta potential [43]. Figure 3A shows changes in the zeta potential of protein from samples with different fat levels and multiple F–T cycles. All samples exhibited a negative charge, which was attributed to negatively charged amino acid residues (glutamic and aspartic acids) [34,44]. When the number of F–T cycles increased to five, the absolute value of protein zeta potential significantly decreased by 46.18% in samples with 20% fat (*p* < 0.05). The lower absolute zeta potential could be relevant to larger particle size in samples with 20% fat, thus leading to particle agglomeration between proteins. The results could be explained by the fact that the partial unfolding of proteins and protein aggregation during F–T cycles led to an imbalance in the proportion of acids and basic amino acids, which led to the change in net surface charge [45]. Vate and Benjakul [46] reported that proteins with lower absolute zeta potential had a smaller repulsive force between particles, which made aggregation easier. Furthermore, the formation of aggregate influenced the surface charge of proteins. Shen et al. [36] reported that the generation of a number of polymers due to the instability of protein solution reduced the protein molecules’ exposed surface charges. The results agreed with the observed change in protein solubility (Figure 1B), which confirmed that F–T treatments and fat levels increased the instability of protein solutions.

#### 3.2.3. Protein Turbidity

Turbidity changes are important for evaluating the state of protein aggregation [24]. As shown in Figure 3B, as the number of F–T cycles increased, the turbidity of samples significantly increased at the same fat level (*p* < 0.05). The results could be explained by the fact that protein conformation unfolding due to the exposure of hydrophobic residues during F–T cycles could promote protein–protein interactions and the formation of large protein aggregates [3]. Wang et al. [40] reported that the increased turbidity could be explained by the fact that the monomeric protein converted to a number of macromolecular proteins by polymerization. Li et al. [47] concluded a similar result that the destruction of protein conformation and exposure of hydrophobic groups led to the formation of high-molecular-weight aggregates, which then led to an increase in protein turbidity. Sow et al. [48] obtained similar results that the unfolding of protein structure could facilitate aggregation, which resulted in an increase in turbidity.

When samples underwent the same number of F–T cycles, the turbidity of MP significantly increased as fat levels increased (*p* < 0.05). For samples with 20% fat at the fifth F–T cycle, the changes in turbidity significantly increased by 20.93% compared with those in patties without fat. The results could be explained by the fact that lipid oxidation could have aggravated the extent of protein oxidation during F–T cycles, which induced protein denaturation and promoted the formation of large aggregates, thus increasing the turbidity of MP suspensions [36]. The turbidity differences from different fat levels might be related to the disparity in the rate and degree of protein aggregation, as samples with higher fat levels possibly formed large protein aggregates. The turbidity of MP suspension could be confirmed with an increase in surface hydrophobicity (Figure 1A) and a decrease in protein solubility (Figure 1B).

### 3.3. Emulsifying Property

#### 3.3.1. EAI and ESI

**Figure 4:**
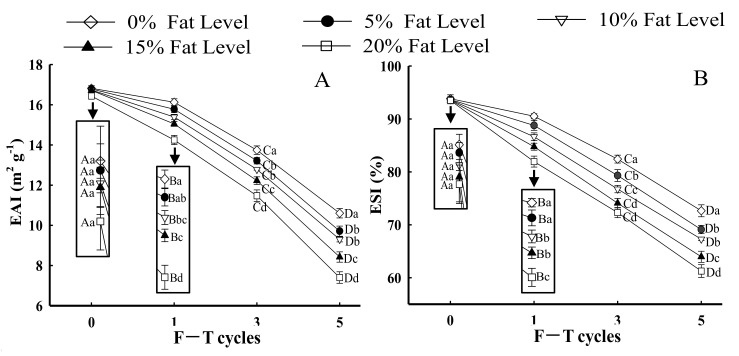

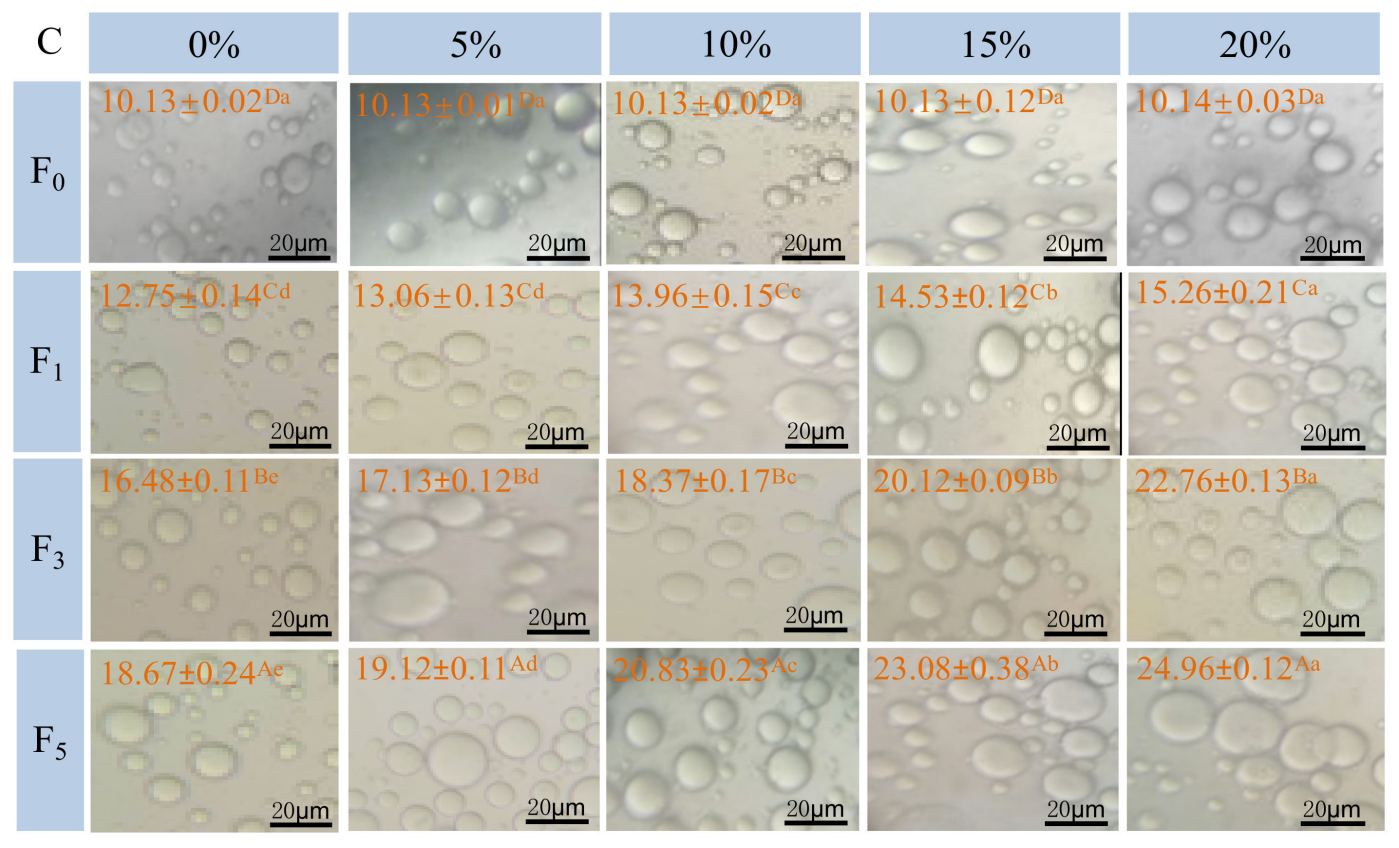
Influence of F–T cycles on emulsifying activity index (EAI) (**A**), emulsifying stability index (ESI) (**B**), the diameter of myofibrillar protein emulsion droplets (μm) and the distribution (**C**) of myofibrillar protein from quick-frozen patties with different fat levels. The magnification was set as × 40. Values are given as the mean ± SE. The means for the same fat levels. with different uppercase letters (A to D) and the same F–T cycles with different lowercase letters (a to e) differ significantly (*p* < 0.05).

The emulsifying property shows the ability of protein to adsorb at the oil–water interface, which can be evaluated using EAI and ESI [24]. As depicted in Figure 4A,B, the EAI and ESI of MP from samples with the same F–T cycles were significantly lesser with greater levels of fat (*p* < 0.05). The reduction in EAI and ESI of MP could be explained by the fact that the formation of intra-/intermolecular disulfide bonds and oxidative reactions induced by F–T processes led to a less stable protein conformation, which prevented the formation of stable emulsions [49,50]. Moreover, when the samples with 20% fat levels were at the fifth F–T cycle, the EAI and ESI of MP significantly declined by 26.73 and 14.15%, respectively, compared with those of MP from patties without fat. The results could be attributed to proteins from patties with higher amounts of fat being more prone to denaturation and easily forming large insoluble aggregates. Note that the formation of large protein aggregates caused fewer protein molecules to be adsorbed at the oil–water interface, which led to a decrease in the EAI and ESI of MP [51]. Furthermore, Zhang et al. [20] demonstrated that aggregates might reduce the flexibility of protein to adsorb at the surface of lipid droplets, thus decreasing emulsifying properties. The result was associated with a change in particle size (Figure 2) in which a larger particle size had a negative influence on emulsifying ability and stability.

#### 3.3.2. Microstructure of Emulsion

Figure 4C shows optical micrographs of emulsion droplet distribution and size prepared with different samples under F–T cycles. The droplets of fresh samples exhibited a relatively small and homogeneous shape, and the average diameter of droplets was 10.13 μm. As fat levels and the number of F–T cycles increased, larger oil droplets were formed. Moreover, oil flocculation, dense and irregular distribution of emulsion droplets, was observed. The droplet size was increased to 18.67 (0%), 19.12 (5%), 20.83 (10%), 23.08 (15%), and 24.96 μm (20%) after five F–T cycles. The phenomenon agreed with the change in particle size (Figure 2) and emulsifying properties (Figure 4A,B). The results could be explained by the fact that freezing and thawing processes, along with oxidative reactions, decreased the emulsifying properties of the protein and promoted the formation of aggregates between protein molecules [51].

### 3.4. Correlation Analysis

**Figure 5:**
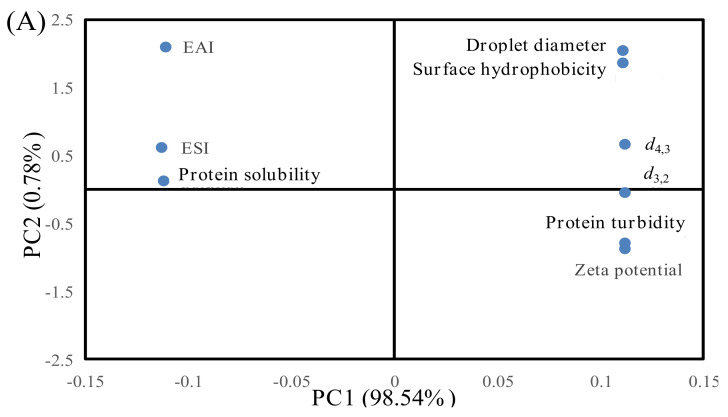

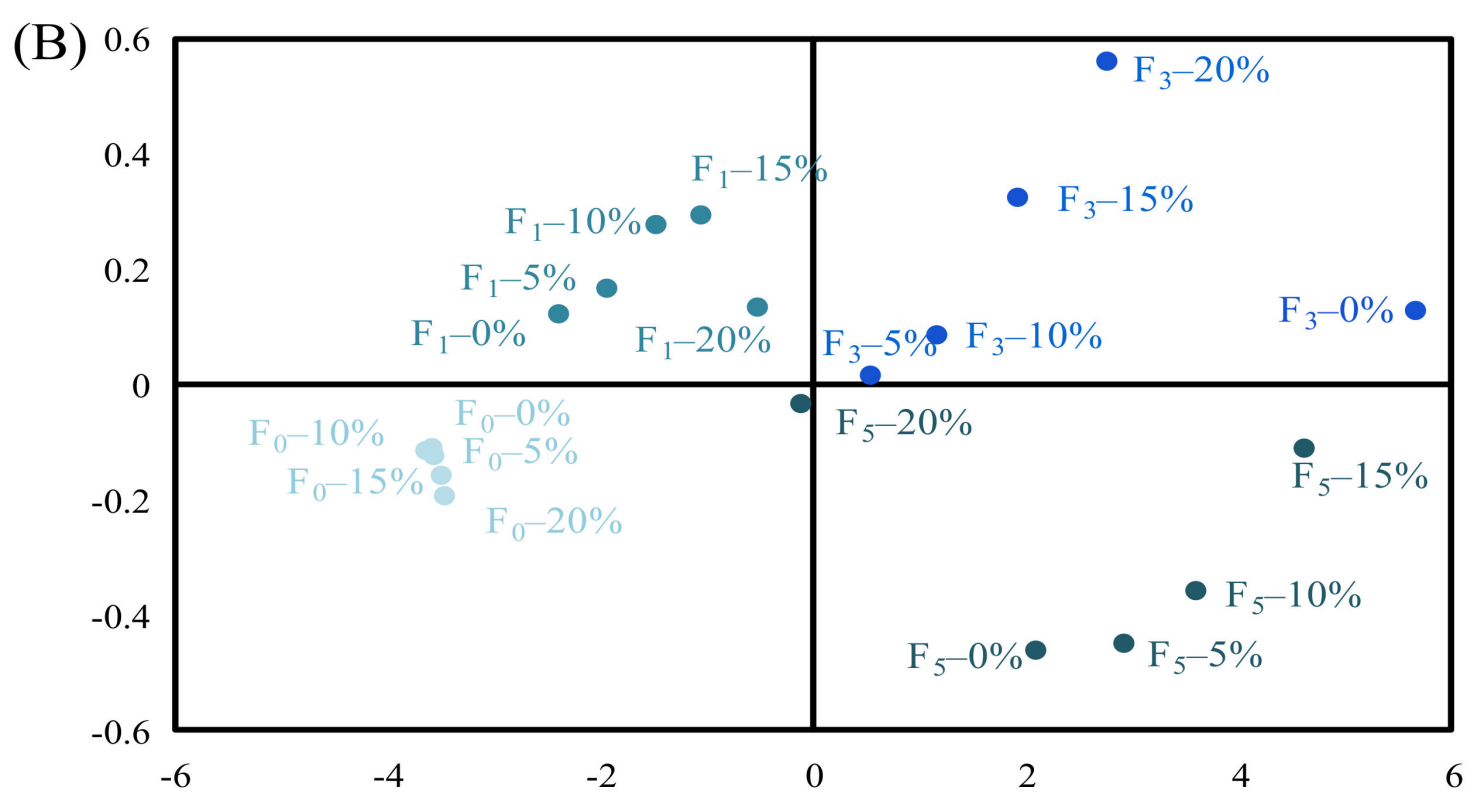
Principal component analysis (PCA) of the indicators and patties at different F–T cycles. Weighed PCA biplot of (**A**) factor loading plot and (**B**) factor scores plot for the first two principal components, accounting for approximately 99.32% of the total variance of the data. PC1 describes 98.54% of the variation, and PC2 explains 0.78%.

PCA analysis was performed to establish the correlations between the indicators (surface hydrophobicity, protein solubility, *d*_4,3_, *d*_3,2_ zeta potential, protein turbidity droplet diameter, EAI and ESI) as well as between the indicators and different F–T cycles (as shown in Table 2 and Figure 5).

As shown in Table 2, correlation analysis showed that the surface hydrophobicity was negatively related to EAI and ESI and that this appeared to be extremely significant correlation (*p* < 0.01). Protein solubility was positively related to EAI and ESI. Xia et al. [27] indicated that protein with higher surface hydrophobicity could lower the protein solubility and promote protein denaturation and aggregation. The formation of protein aggregates caused fewer protein molecules to be adsorbed at the oil–water interface, leading to a decrease in the EAI and ESI of MP. Moreover, *d*_4,3_, *d*_3,2_, droplet size, turbidity, and zeta potential were extremely correlated with EAI and ESI (*p* < 0.01). An increase in the particle size and protein turbidity might exacerbate protein–protein interactions and facilitate the formation of large protein aggregates [3]. The formation of aggregates might lower the protein capacity to adsorb at the surface of lipid droplets, thus resulting in a decrease in emulsifying ability [20].

Figure 5 shows score plots for the principal components (PC1 and PC2), the first two principal components, which explained about 98.54% and 0.78% of the overall variance, respectively. The changes in protein properties can be inferred to be located in designated quadrants 1, 2, 3, and 4. As shown in Figure 5A, EAI, ESI, and protein solubility of samples were distributed in the negative part of PC1, while droplet diameter, surface hydrophobicity, *d*_4,3_, *d*_3,2_, protein turbidity, and zeta potential were distributed in the positive side of PC1, indicating that EAI and ESI were positively correlated with protein solubility but negatively correlated with droplet diameter, surface hydrophobicity, *d*_4,3_, *d*_3,2_, protein turbidity, and zeta potential. The results are also shown in Table 2. Figure 5B shows differentiations among the samples under different F–T cycles. The samples could be separated into two groups. The first group consisted of F_0_ (0%, 5%, 10%, 15%, and 20%) and F_1_ (0%, 5%, 10%, 15%, and 20%) samples. The two samples were distributed in the left quadrant, and they were positively correlated with EAI, ESI, and protein solubility. When the samples were in the F_0_ and F_1_ cycles, the damage to protein structure was relatively small. Thus, protein had a relatively high solubility and could be adsorbed at the oil–water interface. The second group consisted of F_3_ (0%, 5%, 10%, 15%, and 20%) and F_5_ (0%, 5%, 10%, 15%, and 20%) samples. The two samples were distributed in the right quadrant, and they were positively correlated with droplet diameter, surface hydrophobicity, *d*_4,3_, *d*_3,2_, protein turbidity, and zeta potential. When patties were subjected to repeated F–T cycles, protein conformation was unfolded, and certain hydrophobic groups were exposed to the polar environments of the protein, which subsequently participated in the processes of protein aggregation. As a result, the degree of protein denaturation and aggregation was increased with increased numbers of F–T cycles, and the effects on protein turbidity and zeta potential were more significant at the fifth F–T cycle.

### 3.5. Possible Mechanism Schematic for Decreased Emulsifying Performance

**Figure 6:**
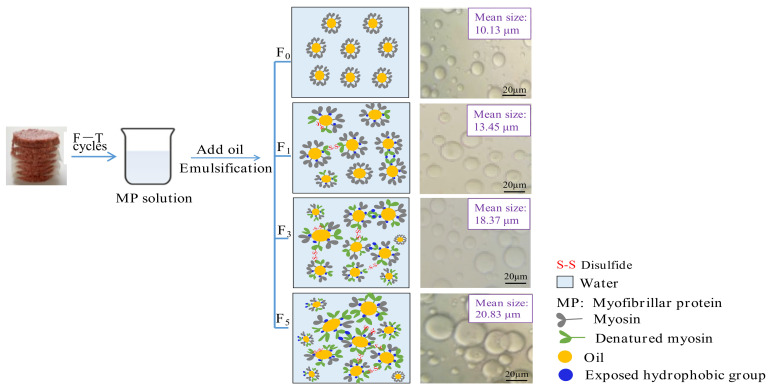
Proposed schematic illustration for the decreased emulsifying property of protein during F–T cycles caused by protein aggregation and denaturation.

The decrease in the MP emulsifying property because of protein aggregation and denaturation was demonstrated by the aforementioned results (as shown in Section 3.1, Section 3.2 and Section 3.3) in our study. Furthermore, a schematic was proposed and is exhibited in Figure 6. Myosin is the major protein in myofibrils and has a relatively strong ability to adsorb on emulsion droplets [2]. For the F_0_ samples, myosin in solution positioned the head of the aqueous phase and positioned the rod-like tail in the oil phase. The emulsion droplets were evenly distributed in the solution, and shape was homogeneous. During freezing and thawing processes, myosin gradually unfolded, which was followed by the subsequent exposure of buried polar and hydrophobic groups. Consequently, the sulfhydryl groups in the head of myosin were easily oxidized, which triggered the formation of protein aggregates [11]. The aggregate formation reduced the ability of the protein to adsorb at the oil–water interface, causing a reduction in emulsifying performance. Furthermore, the increase in the content of denatured protein triggered irregular distribution and flocculation of emulsion droplets, which caused a reduction in the emulsifying property of MP.

## 4. Conclusions

With increased fat levels and F–T cycles of patties, protein denaturation and aggregation increased, which was demonstrated by increases in surface hydrophobicity, turbidity, and particle size, in addition to reductions in solubility and the absolute zeta potential of proteins. Patties with higher fat levels could aggravate the extent of protein denaturation and aggregation, which thus weakened the emulsifying property in terms of reduced EAI and ESI and the increased size of emulsion droplets. To summarize, protein denaturation and aggregation demonstrated a negative impact on the emulsifying property of quick-frozen patties after multiple F–T cycles.

## Figures and Tables

**Table 1:** Influence of F–T cycles on particle size (*d*_4,3_ and *d*_3,2_) of myofibrillar protein from quick-frozen patties with different fat levels.

Particle Size (μm)	F–T Cycles	Fat Levels (%)				
		0	5	10	15	20
*d*_4,3_	F_0_	41.29 ± 0.21 ^Da^	41.25 ± 0.22 ^Da^	41.36 ± 0.21 ^Da^	41.45 ± 0.37 ^Da^	41.33 ± 0.37 ^Da^
	F_1_	44.19 ± 0.61 ^Cc^	45.39 ± 0.41 ^Cb^	46.13 ± 0.14 ^Cab^	46.76 ± 0.28 ^Ca^	47.05 ± 0.53 ^Ca^
	F_3_	47.45 ± 0.51 ^Bd^	48.52 ± 0.41 ^Bc^	49.61 ± 0.31 ^Bb^	50.95 ± 0.09 ^Ba^	51.61 ± 0.25 ^Ba^
	F_5_	50.49 ± 0.43 ^Ad^	52.49 ± 0.43 ^Ac^	53.49 ± 0.43 ^Ac^	54.54 ± 0.27 ^Ab^	56.54 ± 0.26 ^Aa^
*d*_3,2_	F_0_	12.32 ± 0.17 ^Da^	12.27 ± 0.19 ^Da^	12.21 ± 0.14 ^Da^	12.23 ± 0.21 ^Da^	12.21 ± 0.14 ^Da^
	F_1_	14.62 ± 0.31 ^Cd^	15.23 ± 0.21 ^Ccd^	15.66 ± 0.24 ^Cbc^	16.19 ± 0.07 ^Cab^	16.69 ± 0.24 ^Ca^
	F_3_	17.29 ± 0.12 ^Be^	17.78 ± 0.14 ^Bd^	18.34 ± 0.08 ^Bc^	18.81 ± 0.18 ^Bb^	19.35 ± 0.23 ^Ba^
	F_5_	19.53 ± 0.21 ^Ae^	20.51 ± 0.17 ^Ad^	21.61 ± 0.12 ^Ac^	22.37 ± 0.23 ^Ab^	23.38 ± 0.26 ^Aa^

Values are given as the mean ± SE. The means for the same fat levels with different uppercase letters (A–D) and the same F–T cycles with different lowercase letters (a to e) differed significantly (*p* < 0.05).

**Table 2:** Correlations among protein denaturation, protein aggregation, and the emulsifying property of MP from samples with different fat levels and F–T cycles.

	Surface Hydrophobicity	Protein Solubility	*d*_4,3_	*d*_3,2_	Zeta Potential	Protein Turbidity	Droplet Diameter	EAI	ESI
Surface hydrophobicity	1								
Protein solubility	−0.984 **	1							
*d*_4,3_	0.983 **	−0.986 **	1						
*d*_3,2_	0.971 **	−0.983 **	0.997 **	1					
Zeta potential	0.973 **	−0.995 **	0.988 **	0.989 **	1				
Protein turbidity	0.972 **	−0.994 **	0.983 **	0.981 **	0.998 **	1			
Droplet diameter	0.987 **	−0.985 **	0.983 **	0.974 **	0.977 **	0.979 **	1		
EAI	−0.960 **	0.987 **	−0.972 **	−0.976 **	−0.992 **	−0.989 **	−0.951 **	1	
ESI	−0.980 **	0.995 **	−0.991 **	−0.990 **	−0.997 **	−0.995 **	−0.977 **	0.991 **	1

Note: **, extremely significant correlation (*p* < 0.01). Minus signs indicate that the corresponding indicators are negatively correlated; their absence indicates that the corresponding indicators are positively correlated.

## Data Availability

The data presented in this study are available in the article.
